# Occurrence of Esophageal Atresia With Tracheoesophageal Fistula in Siblings From Three-Generation Family Affected by Variable Expressivity *MYCN* Mutation: A Case Report

**DOI:** 10.3389/fped.2021.783553

**Published:** 2021-12-02

**Authors:** Magdalena Klaniewska, Krystian Toczewski, Anna Rozensztrauch, Michal Bloch, Agata Dzielendziak, Piotr Gasperowicz, Ryszard Slezak, Rafał Ploski, Małgorzata Rydzanicz, Robert Smigiel, Dariusz Patkowski

**Affiliations:** ^1^Department of Pediatrics and Rare Disorders, Medical University, Wroclaw, Poland; ^2^Department of Pediatric Surgery and Urology, Medical University, Wroclaw, Poland; ^3^Department of Medical Genetics, Medical University, Warsaw, Poland; ^4^Department of Genetics, Medical University, Wroclaw, Poland

**Keywords:** esophageal atresia, tracheoesophageal fistula, *MYCN*, Feingold syndrome, familial occurrence

## Abstract

The *MYCN* oncogene encodes a transcription factor belonging to the MYC family. It is primarily expressed in normal developing embryos and is thought to be critical in brain and other neural development. Loss-of-function variants resulting in haploinsufficiency of *MYCN*, which encodes a protein with a basic helix–loop–helix domain causes Feingold syndrome (OMIM 164280, ORPHA 391641). We present an occurrence of esophageal atresia (EA) with tracheoesophageal fistula in siblings from a three-generation family affected by variable expressivity of *MYCN* mutation p.(Ser90GlnfsTer176) as a diagnostic effect of searching the cause of familial esophageal atresia using NGS-based whole-exome sequencing (WES). All of our affected patients showed microcephaly and toe syndactyly, which were frequently reported in the literature. Just one patient exhibited clinodactyly. None of the patients exhibited brachymesophalangy or hypoplastic thumbs. The latest report noted that patients with EA and Feingold syndrome were also those with the more complex and severe phenotype. However, following a thorough review of the present literature, the same association was not found, which is also confirmed by the case we described. The variable phenotypic expression of the patients we described and the data from the literature guide a careful differential diagnosis of Feingold syndrome even in cases of poorly expressed and non-specific symptoms.

## Introduction

Esophageal atresia (EA) is a rare and severe congenital anomaly characterized by discontinuity of the esophagus with or without a persistent communication with the trachea. In 86% of the cases, there is a distal tracheoesophageal fistula. The etiology of EA is unclear, and its pathogenesis is controversial. Recent large studies have identified the prevalence of EA to be 2.3–2.4 cases per 10,000 births. In the last decade, survival has increased to 91–98%. Of patients with congenital EA, 45% present an isolated form without any additional associated defects. In the remaining 55% of patients, EA coexists with other defects (syndromic form), which, together, may form a genetic syndrome or association (1, 2).

Esophageal atresia is one of the malformations found in Feingold syndrome (1). Feingold syndrome (OMIM 164280, ORPHA 391641) is a rare disease originally described by Feingold in 1975, characterized by autosomal dominant inheritance of digital anomalies, microcephaly, facial dysmorphism, gastrointestinal atresias, and learning disability. It is caused by loss-of-function variants resulting in haploinsufficiency of *MYCN*, which encodes a protein with a basic helix–loop–helix domain. To date, more than 120 patients have been described in literature (3).

### Case Description

We present an occurrence of esophageal atresia with tracheoesophageal fistula in siblings from a three-generation family affected by variable expressivity of *MYCN* p.(Ser90GlnfsTer176) mutation. For the majority of previously reported families, the condition is unique to one child, which, in itself, is challenging.

As EA is uncommon in the general population, it is extremely rare to affect more than one sibling in any family. The risk of recurrence is 0.5–2% and rises to 20% if another sibling is affected (4). NGS-based whole-exome sequencing (WES) was used to establish the genetic cause of familial EA in our patients.

### Family Description

**Figure 1:**
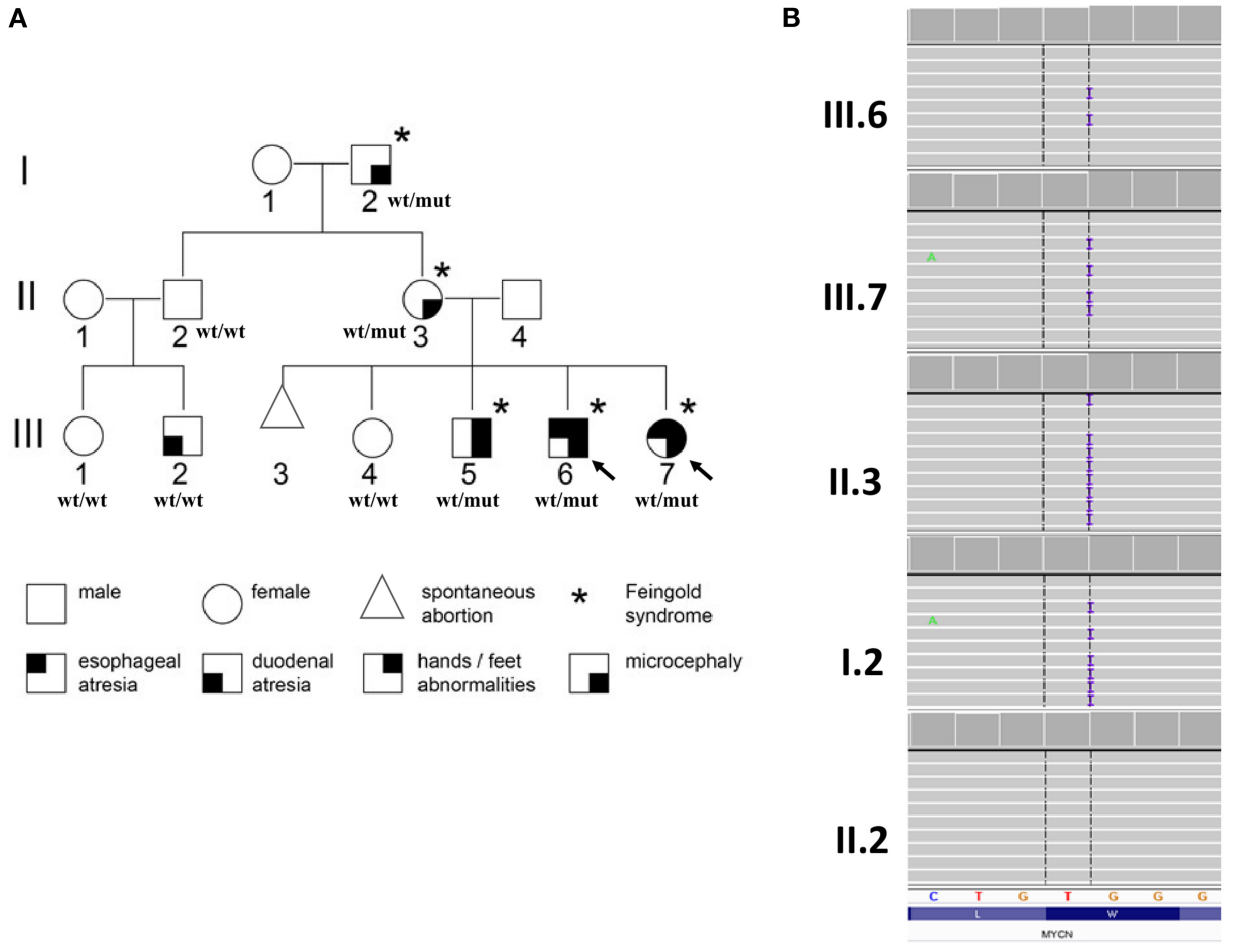
Pedigree of the examined family with phenotype–genotype information **(A)** and partial results of genetic evaluation performed by amplicon deep sequencing **(B)**. Arrows indicate probands for whom whole-exome sequencing was performed; wt/wt, wild-type genotype; wt/mut, carries of heterozygous p.(Ser90GlnfsTer176) variant in *MYCN* gene.

A three-generation family with Feingold syndrome was clinically and genetically evaluated (Figure 1).

### Patient 1

Child III.6 is a boy born to non-consanguineous parents from gravida IV para III. It was a spontaneous vaginal delivery at the 38th week of uneventful pregnancy, and the birth weight was 3,550 g. Esophageal atresia with distal tracheoesophageal fistula (TEF) was diagnosed (Gross type C). In the second day of life, the patient underwent thoracoscopic esophageal atresia repair.

The postoperative period according to our local protocol was uneventful, and the boy was discharged home on the 14th day of life. On the 13th and 15th months of life, the patient had moderate anastomotic stricture treated with endoscopic dilations. The child remained under constant care of our pediatric surgery clinic and a speech therapist. Until the age of 4 years, the parents occasionally observed choking, but this was resolved. At the time of writing of this manuscript, the patient is 5 years old and free from feeding difficulties.

**Figure 2:**
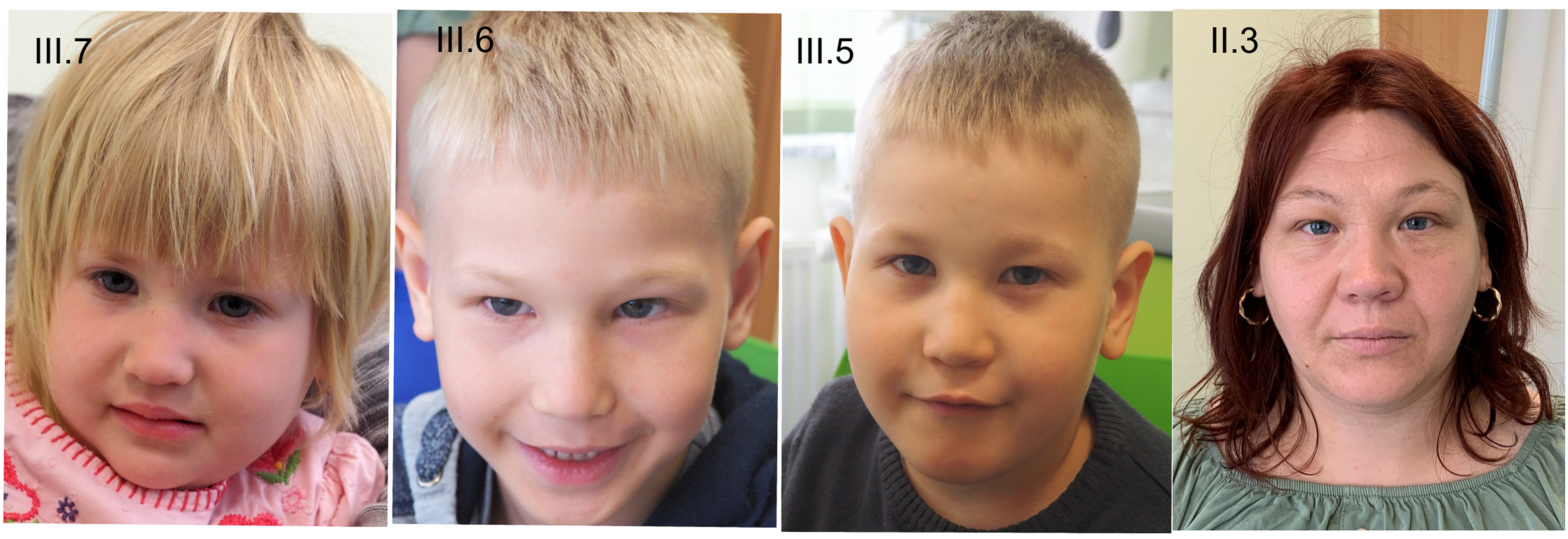
Phenotype of the patients. III.7−3-year-old girl with EA, III.6−5-year-old boy with EA, III.5−9 years old boy, II.3—mother.

**Figure 3:**
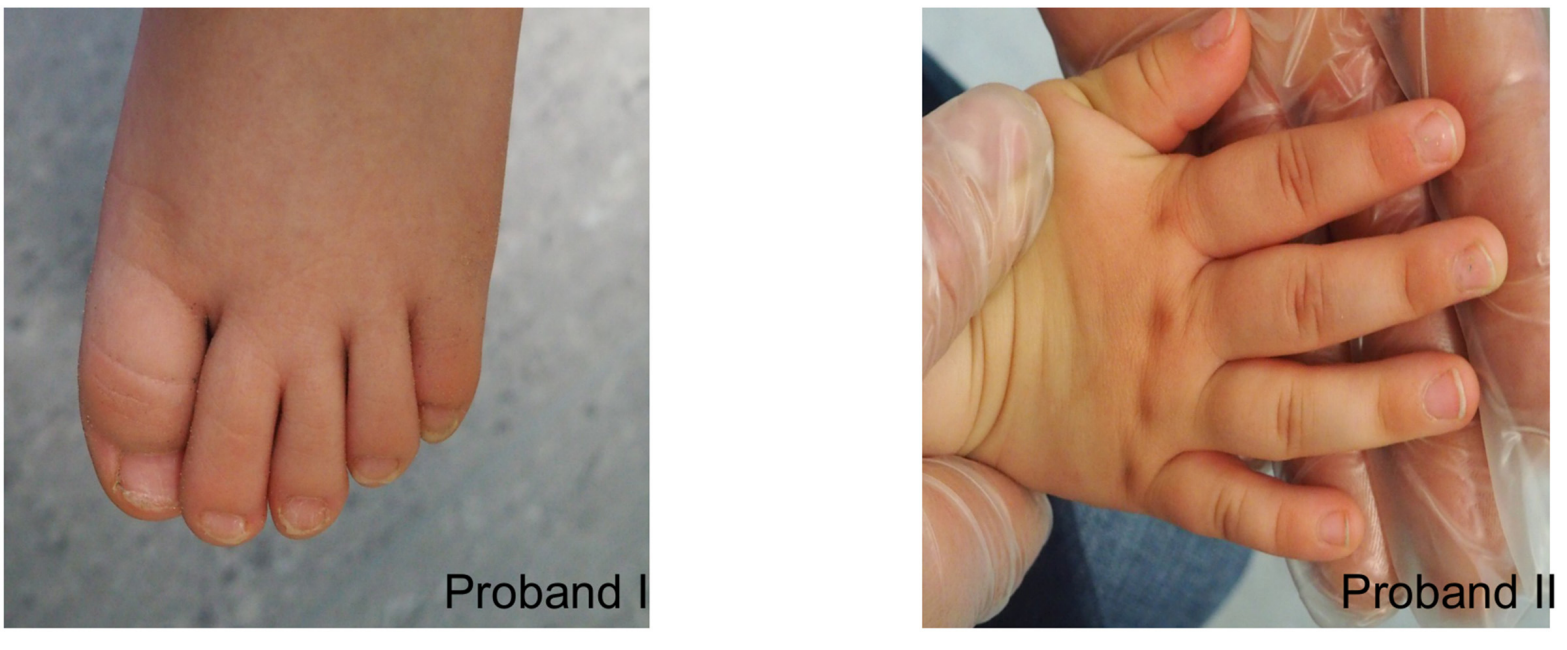
Syndactyly of II and III toes in proband I. Mild clinodactyly of fingers IV and mild skin syndactyly of II and IV fingers in proband II.

At physical examination (at 5 years), his height is 110 cm (55pc), weight 24 kg (97pc), and his head circumference (OFC) is 47 cm (<1pc) (Figure 2). Additionally, he has a mild syndactyly of II and III toes (Figure 3), but his hands are normal. His mental development is impaired.

### Patient 2

Almost 2 years after the abovementioned child, a girl (III.7) was born to the same parents. She was delivered vaginally at the 42nd week of pregnancy to gravida V para IV. Her birth weight was 3,300 g, and she scored an Apgar 10. On prenatal ultrasound, a polyhydramnios was diagnosed. After birth, PFO and VSD were diagnosed but resolved spontaneously. Like her older brother, on the second day of life, she underwent esophageal atresia type C repair by thoracoscopy.

The postoperative course was smooth. An anastomotic stricture was dilated three times (at the 14th, 20th, and 23rd months of life). At the 27th month of life, she underwent endoscopy for a food bolus stuck in the esophagus. The esophagoscopy showed no evident stenosis. At the age of 3 years, a control gastroscopy did not reveal any pathology. She suffers from mild transient difficulties with swallowing solid foods.

At 3 years old, she is 93 cm tall (27pc), weighs 14 kg (51pc), and her OFC is 44.5 cm (<1pc) (Figure 2). Moreover, mild clinodactyly of fingers IV and mild skin syndactyly of II and IV fingers (Figure 3) as well as mild syndactyly of II and III toes were observed. Her cognitive development is quite good.

### Other Members of the Family

Because of the esophageal atresia in the siblings, the whole family underwent genetic counseling. The parents have two more children, and there is a history of one spontaneous abortion. The oldest child (III.4), a 10-year-old girl, is healthy and develops in normal range. In the second oldest child (III.5), a 9-year-old boy, developmental delay and intellectual disability were diagnosed. The boy started walking at 3 years, and speech developed at 5 years. Behavioral symptoms such as hyperactivity and attention deficit were also observed. Moreover, the boy presents syndactyly of II and III toes. At the age of 9 years, he is 123 cm tall (5 percentile), weighs 25 kg (20 percentile), and has an OFC of 48 cm (<1 percentile) (Figure 2). Both older siblings are not affected by esophageal atresia. The only observed symptom in the mother (II.3) is microcephaly (OFC 53 cm, 10–25 percentile) (Figure 2). She graduated from a vocational school. The microcephaly was observed also in the father of the mother (I.2; OFC 53 cm, 3–10 percentile). His intellect is borderline; he manages to work as farmer and tractor driver.

The brother of the mother (II.2) is a healthy father to two children. His son (III.2) was operated on for duodenal atresia but the OFC of the child is normal (51 cm at age of 4 years, 25–50 percentile).

### Genetics Study and Results

Whole-exome sequencing (WES) was performed on the probands [III.6 and III.7 (Figure 1A)] in order to establish the cause of familial EA. DNA purified from whole blood was used for library preparation performed using SureSelectXT Human kit All Exon v7 (Agilent, Agilent Technologies, Santa Clara, CA, USA). Enriched libraries were paired-end sequenced (2 × 100 bp) on NovaSeq 6000 (Illumina, San Diego, CA, USA). Raw NGS data analysis and variant prioritization were performed as previously described (5). Variants considered as causative were further validated in the probands and studied in all available family members (I.2, II.2, II.3, III.1, III.2, III.4, III.5) by amplicon deep sequencing (ADS) performed using Nextera XT Kit (Illumina) and paired-end sequenced (2 × 100 bp) on HiSeq 1500 (Illumina).

In both probands, shared heterozygous frameshift variant in exon 2 of *MYCN* gene was prioritized [hg38, chr2:g.15942327, NM_005378.6:c.266dupG/p.(Ser90GlnfsTer176)]. The p.(Ser90GlnfsTer176) has 0 frequency in all tested databases and is predicted by NMDEscPredictor (6) degraded by non-sense-mediated decay; thus, reduction of *MYCN* expression is expected.

The performed family study showed that affected maternal grandfather (I.2), mother (II.3) (Figure 1B), and older brother (III.5) (data not shown) of the probands harbor the *MYCN* p.(Ser90GlnfsTer176) variant, while unaffected family members, II.2 (Figure 1B), III.1, III.2, and III.4 (data not shown) are wild type. The observed variant segregation with the disease within the examined family is consistent with autosomal dominant pattern of inheritance. No other variant(s) was revealed by WES corresponding to clinical phenotype, autosomal dominant model of inheritance predicted by pedigree analysis or fulfilled frequency, and pathogenicity criteria of gene variants.

## Discussion

Congenital esophageal atresia may coexist with at least seven identified genetic disorders. These include associations such as VATER/VACTERL, as well as CHARGE syndrome, Feingold syndrome, anophthalmia–esophageal–genital (AEG) syndrome, Pallister–Hall syndrome, Opitz syndrome, and Fanconi anemia. In recent years, the key genes for these diseases have been identified.

Murray Feingold in 1975 described a boy with tracheoesophageal fistula type C, duodenal atresia, microcephaly, micrognathia, apparent absence of middle phalanx of the fifth fingers, bilateral syndactyly of toes 2–3, narrow palpebral fissures, rocker bottom feet, and overlapping toes. The presented father and paternal grandmother of the patient, in the mentioned manuscript, also had microcephaly and the same hand and foot abnormalities. They were said to be of normal intelligence (7).

The Feingold syndrome has been mapped to 2p23–242 and is the consequence of *MYCN* gene loss-of-function either by germline deletions or by coding-sequence mutations (8). Although the condition is autosomal dominant, 63% of the patients are female (9). The protein product of *MYCN* gene is located in the nucleus and is involved in transcriptional regulation, cell cycle, differentiation, and morphogenesis, being under the control of the SHH, WNT, TGF, and FGF signaling pathways. Up until now, 23 different point mutations as well as five deletions in the *MYCN* gene have been identified. Approximately 30% to 40% of patients with Feingold syndrome have EA and TEF (10).

Significant and interesting in the family described in this paper is not so much the occurrence of Feingold's syndrome itself in three generations, but the fact that siblings present EA, where the chance of recurrence of EA in the family is low and amounts to 0.2–2%. On the other hand, EA occurs as a symptom of Feingold's syndrome between 30 and 40% of cases according to the literature. Therefore, we can conclude that this combination is quite rare and worth presenting.

It is also important to emphasize the nature of phenotypic variability occurring in our described familial case of Feingold's syndrome. The relatively marked expression of symptoms in the siblings, although phenotypically variable, correlates with poorly expressed clinical symptoms in previous generations. The only symptom observed in the mother of the sibling and the father of the mother was microcephaly, consequently, causing a lower level of intellectual functioning but within normal range. The example we describe here shows some analogy to the case of Balumeiser, who, with his team, reported monozygotic female twins with Feingold syndrome associated with a mutation in the *MYCN* gene. Interestingly, phenotypic variability was observed among twins. Family history and genetic analysis showed that the mother and maternal grandfather also carried the mutation, but had only finger and toe anomalies (11).

Marcelis et al. in 2008 reviewed the clinical features of 77 patients with mutations and compared them with the largest previous review (12), finding that digital anomalies including brachymesophalangia and finger syndactyly were the most consistent features, present in 100% and 97% of the patients, respectively. Interestingly, none of the patients we described presented brachymesophalangia, and syndactyly appeared only in affected children. In contrast, small head circumference was reported by Marcelis in 89% of the cases, whereas in the family we described, microcephaly affected all diagnosed members. According to the review of Marcelis, gastrointestinal atresia was the most important major congenital anomaly (55%), but renal and cardiac anomalies were also common (18 and 15%, respectively) (13). In contrast, facial dysmorhpism includes short palatal fissures (73%) and micrognathia (30%) (7). Learning disabilities are common in Feingold syndrome, but most patients are able to lead independent lives. However Tedesco et al. (2021) described the first ever patient with severe intellectual disability having a maternally inherited *MYCN* variant together with an additional *GNAO1* mutation inherited paternally (3).

Our report and previously published data clearly demonstrate intra- and interfamilial phenotypic variability of *MYCN* mutations, including in monozygotic twins. It has been suggested that in patients with a more complex phenotype, in addition to mutations in *MYCN*, coexisting variants in other genes and/or environmental factors may contribute to the observed variability. However, neither our study nor previous reports provide sufficient evidence to support this hypothesis.

The variable phenotypic expression of the patients we described, as well as the literature data, may also prompt us to lean toward a careful differential diagnosis of Feingold syndrome in the presence of poorly expressed and non-specific symptoms.

In addition, we believe that the description of the cases we have reported can make the medical community be aware of the necessity of genetic counseling for all patients affected by EA (with or without associated abnormalities) and their families. It is extremely important for genetic counseling to be widely provided in this area. We are convinced that this will bring a new diagnostic quality, especially for possible genetic coexisting diseases with poor phenotypic expression and, thus, in the long run, will significantly improve the quality of life of patients and their families.

## Data Availability Statement

The datasets presented in this study can be found in online repositories. The names of the repository/repositories and accession number(s) can be found here https://databases.lovd.nl/shared/genes%20Individual%20accession%20#00386956.

## Ethics Statement

Ethical review and approval was not required for the study on human participants in accordance with the local legislation and institutional requirements. Written informed consent to participate in this study was provided by the participants' legal guardian/next of kin. Written informed consent was obtained from the individual(s), and minor(s)' legal guardian/next of kin, for the publication of any potentially identifiable images or data included in this article.

## Author Contributions

MK, RSm, and KT were responsible for the case report. MR and PG were responsible for the molecular analysis. MK, KT, AR, MB, AD, and RSl were responsible for the manuscript writing and literature review. RSm, DP, and RP were responsible for the manuscript revising. The authors alone are responsible for the content and writing of this article. All authors have read and approved the final manuscript.

## Funding

This research was supported by the Ministry of Health subvention according to number STM.E164.20.059 from the IT Simple system of Wroclaw Medical University.

## Conflict of Interest

The authors declare that the research was conducted in the absence of any commercial or financial relationships that could be construed as a potential conflict of interest.

## Publisher's Note

All claims expressed in this article are solely those of the authors and do not necessarily represent those of their affiliated organizations, or those of the publisher, the editors and the reviewers. Any product that may be evaluated in this article, or claim that may be made by its manufacturer, is not guaranteed or endorsed by the publisher.
